# Changes in Human Colonic Microbiota Promoted by Synbiotic Açai Juice Composed of Gluco-Oligosaccharides, Dextran, and *Bifidobacterium breve* NRRL B-41408

**DOI:** 10.3390/foods13244121

**Published:** 2024-12-20

**Authors:** Bianca Mara Reges, Francisca Andréa da Silva Oliveira, Thatyane Vidal Fonteles, Sueli Rodrigues

**Affiliations:** 1 Food Engineering Department, Federal University of Ceara, Fortaleza 60440-900, CE, Brazil; biancamara@alu.ufc.br (B.M.R.); sueli@ufc.br (S.R.); 2 Drug Research and Development Center, Federal University of Ceara, Fortaleza 60356-001, CE, Brazil; andreasilvaoli@gmail.com

**Keywords:** synbiotic açai juice, functional juices, human fecal microbiota

## Abstract

The present study evaluates the effects of açai juice containing gluco-oligosaccharides and dextran, fermented by *Bifidobacterium breve* NRRL B-41408 (synbiotic juice), on the human fecal microbiota. The juice is subjected to simulated digestion and fecal fermentation after production and 42 days of refrigerated storage. High throughput 16S rRNA sequencing and HPLC are used to identify the bacterial cells and metabolites. The results show that the viability of *B. breve* is stable during the refrigerated storage, indicating that the metabolism is maintained even under low temperatures and pH. Furthermore, gluco-oligosaccharides and dextran prove to be resistant to gastrointestinal conditions and are quickly consumed during fecal fermentation. The synbiotic açai juice enhances the microbial diversity and stimulates the production of short-chain fatty acids (SCFA), including acetate, propionate, and isobutyrate. Elevated propionate levels are directly associated with an increased abundance of *Bacteroides thetaiotaomicron*, *Bacteroides uniformis*, *Bacteroides xylanisolvens*, *Bacteroides dorei, Bacteroides stercoris*, and *Bacteroides massiliensis* after 48 h of fermentation. This highlights the potential of synbiotic açai juice as a functional beverage, supported by the significant increase in microbial diversity reflected in the Shannon and Simpson’s diversity indexes (Shannon = 116.6%, 117.2%, 125.15%, and 116.02%; Simpson’s = 151.86%, 177.22%, 152.5%, and 163.73%).

## 1. Introduction

According to the International Scientific Association for Probiotics and Prebiotics (ISAPP), a synbiotic is “a mixture comprising live microorganisms and substrate(s) selectively utilized by host microorganisms that confers a health benefit on the host”. A synbiotic in which the co-administered microorganisms selectively utilize the substrate is named synergistic. Complementary synbiotics are composed of a probiotic combined with a prebiotic designed to target autochthonous microorganisms. Probiotics and prebiotics must meet the minimum criteria for both components of a complementary synbiotic [1].

Introducing probiotics into the diets of non-dairy consumers can be a challenge, as they are primarily found in dairy products, resulting in an increasing demand for non-dairy functional foods that have spurred the search for new synbiotic options. However, researchers have explored non-dairy matrices, such as fruit juices, to address this issue and develop probiotic foods [2]. Although some technological hurdles need to be overcome, several fruit juices, including açai, cashew apple, melon, apple, jujube, kiwi, and sapota-do-Solimões, have shown promising potential as probiotic vehicles [2,3,4,5,6,7]. In addition, fruit juices can be fortified with prebiotics, which are selectively used by host microorganisms and can provide health benefits such as reducing the risk of obesity and improving the human fecal immune system [8,9]. Sapota-do-Solimões synbiotic juice (fructo-oligosaccharides + *Lacticaseibacillus casei* B-442), for instance, improved human fecal microbiota, shown by the Chao1 increase in microbial diversity after 48 h of fecal fermentation, and increased the content of SCFA, such as propionic, isobutyric, and acetic acids after fecal fermentation of the juice for 30 days at 4 °C [10]. *Bifidobacterium longum* CICC 6259, which is present in synbiotic multi-particulate microparticle containing alginate oligosaccharide (AOS) and chitosan oligosaccharide (COS), has been found to increase the content of *Bifidobacterium* and *Lactobacillus* and reduce the content of *Enterococcus* and *Escherichia* in mice gut microbiota [11]. 

One of the challenging aspects of developing synbiotics is to ensure that the probiotics present in the products remain viable throughout their shelf life to deliver their health benefits successfully. However, even if the probiotics remain viable, their effectiveness in the body is not guaranteed. The gastrointestinal tract and resident microbiota can challenge probiotics’ survival and functionality. Therefore, it is crucial to select strains that can survive and thrive in the gut environment [12]. Additionally, prebiotics can increase the survivability of probiotic strains in synbiotic foods through protective effects, especially when stored at low temperatures [7,13]. The combination of probiotic strains and prebiotic ingredients in fruit juices deserves special attention to improve the consumption of viable probiotic cultures during the entire shelf life of the product. Fermented fruit juices containing gluco-oligosaccharides and dextran have been found to promote the growth of *Lacticaseibacillus casei* and *Bifidobacterium breve* [14,15]. However, studies on synbiotic fruit juice fermented by *Bifidobacterium* species are yet to become available.

This study produces a synbiotic açai juice fermented by *Bifidobacterium breve*. Brazil is the largest producer and exporter of açai pulp, with a high potential for profitability in relation to fruit-derived products with added value and healthy food innovation [16,17]. However, the diversity of products derived from açai is still limited [18]. Açai juice is selected as the base matrix for the synbiotic beverage owing to its rich nutritional profile, which includes high levels of antioxidants, polyphenols, and dietary fiber. These compounds have been shown to possess various health-promoting properties, such as anti-inflammatory effects and antioxidant activity [19]. Açai has also been associated with beneficial effects on gut health, making it an ideal candidate for developing a synbiotic product [20]. Additionally, açai juice has gained popularity in functional food markets, making it a relevant choice for scientific research and potential commercial applications. Therefore, exploring the potential of açai juice as a functional beverage, such as a synbiotic drink, can diversify this commodity. *Bifidobacterium*-containing synbiotics remain underexplored despite their significant role in maintaining gut health. *Bifidobacterium* species belong to the human gut microbiota, particularly in the early life, and have demonstrated a robust potential to manage gut dysbiosis. This study seeks to expand the application of *Bifidobacterium* strains in synbiotic formulations, leveraging their compatibility with prebiotic compounds. In this context, investigating the effects of açai juice containing gluco-oligosaccharides and dextran fermented by *Bifidobacterium breve* NRRL B-41408 on human fecal microbiota composition can be an important step toward developing synbiotic fruit juices with bifidobacteria, potentially exerting a positive effect on gut microbiota balance.

In this study, the main objective is to evaluate the effects of a synbiotic açai juice on human gut microbiota composition and metabolites, with the hypothesis that this juice, containing *Bifidobacterium breve* NRRL B-41408, gluco-oligosaccharides, and dextran, could increase the abundance of beneficial bacterial species. *Bifidobacterium* is known to promote the production of beneficial metabolites and improve the metabolic state by increasing acetate and butyrate concentrations [21]. To test this, the symbiotic juice is stored for 30 days and subjected to in vitro digestion followed by fecal fermentation, where its impact on the human fecal microbiota is assessed.

## 2. Materials and Methods

### 2.1. Synbiotic Açai Juice

The açai juice was prepared from açai (*Euterpe oleraceae*) frozen pulp type A (14% of solids *w*/*w*) purchased at the local market (Fortaleza-CE, Brazil) without sugar, additives, or preservatives. The juice was prepared according to the manufacturer’s recommendations, diluting 100 g of pulp into 200 mL of potable water.

To prepare the prebiotic açai juice, the enzyme dextransucrase from *Leuconostoc mesenteroides* B512 F was used to synthesize the gluco-oligosaccharides and dextran directly into the açai juice. Firstly, the sugar concentration was adjusted to 75 g/L of sucrose and 75 g/L of reducing sugars (glucose and fructose) to act as a substrate for enzymatic synthesis. The pH was adjusted to 5.2 with NaOH, and dextransucrase (1 UI/mL) was added to the juice. The enzymatic synthesis was carried out in a 2 L Becker containing 1 L of açai juice at 30 °C for 6 h under magnetic agitation. After the synthesis, the pH was adjusted to 4.2 with citric acid for enzyme inactivation, halting the reaction [15].

The prebiotic açai juice containing gluco-oligosaccharides and dextran was fermented with the probiotic *Bifidobacterium breve* NRRL B-41408 strain to prepare the synbiotic drink. The *B. breve* NRRL B-41408 stock culture (1.5% *v*/*v*) was previously activated in 100 mL bifidobacteria selective media broth composed of peptone 10 g/L, casein 20 g/L, yeast extract 10 g/L, glucose 20 g/L, tomato extract 8.0 g/L, Tween 2.0 g/L, and 10 mL of dibasic potassium phosphate buffer (20 g/L), pH 6.7. The activation was carried out on an incubator shaker (Solab^®^, Piracicaba, Brazil) at 37 °C for 22 h under mechanical agitation (100 rpm) in anaerobiosis. The anaerobiosis was achieved by sparging the medium with N_2_ using an Erlenmeyer flask with a screw cap and packing it with a plastic film.

**Figure 1:**
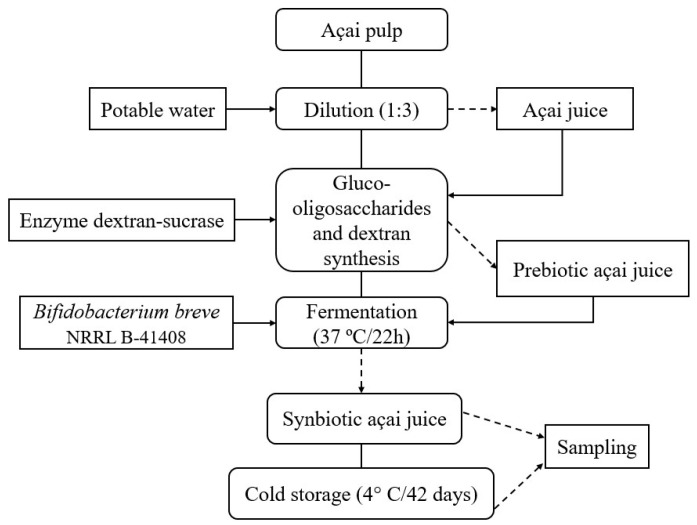
Process flowchart to produce synbiotic açai juice illustrating the dilution of açai pulp, synthesis of gluco-oligosaccharides and dextran using the dextransucrase enzyme, and fermentation with *Bifidobacterium breve* NRRL B-41408 to obtain the synbiotic açai-based beverage.

The prebiotic açai juice was inoculated with 2% (*v*/*v*) of *B. breve* NRRL B-41408 (optical density at 660 nm of 0.5). The inoculum size was based on our findings from previous studies. The initial cell counts were 7.0 log CFU/mL. The juice was incubated statically under anaerobiosis at 37 °C for 21 h in a BOD MA 415 (Marconi^®^, Piracicaba, Brazil). The synbiotic juice was stored at 4 °C for 42 days. The storage period was selected considering the mean shelf-life of probiotics available on the market (30 to 45 days). Also, previous studies carried out by our group indicate that the viability of *B. breve* drops below 6.0 log CFU/mL after 42 days of cold storage. The pH, cell viability, dextran, gluco-oligosaccharides, sugars, and acids were evaluated every 7 days. Figure 1 shows the process flowchart of synbiotic açai juice production. 

### 2.2. Determination of the B. breve NRRL B-41408 Viability

The viability of probiotic cells was assessed in bifidobacteria selective agar, which is composed of agar 15 g/L, peptone 10 g/L, casein 20 g/L, yeast extract 10 g/L, glucose 20 g/L, tomato extract 8.0 g/L, and Tween 2.0 g/L. The viability was expressed as log CFU/mL. Counts were performed in triplicates at 37 °C for 48 h [22]. The microbial viability was assessed after fermentation (day 0) and every 7 days of cold storage up to 42 days. 

### 2.3. B. breve NRRL B-41408 Survival During In-Vitro-Simulated Digestion

The survival of *B. breve* NRRL B-41408 under gastrointestinal conditions was evaluated in a semi-dynamic model of in-vitro-simulated digestion. The system comprised three sequential glass bioreactors (300 mL) coupled with a BIO-TEC-PRO^®^ controller (Tecnal, Piracicaba, Brazil) [14]. The temperature was set to 37 °C, and the sample was subject to 350 rpm agitation.

Gastric and enteric solutions were added to the bioreactors in each digestion phase and prepared according to Lo Curto et al., Martinez et al., and Minekus et al. [23,24,25], adapted by Leite et al. [14]. The gastric phase was simulated using NaCl 47.2 mmol/L, KCl 6.9 mmol/L, KH_2_PO_4_ 0.9 mmol/L, MgCl_2_(H_2_O)_6_ 0.1 mmol/L, NaHCO_3_ 25 mmol/L, CaCl_2_ 0.3 g/L, and pepsin 600 U/mL (2.4 g/L) (Sigma Aldrich, St. Louis, MO, USA). The pH was adjusted with NaOH 1 M or HCl 1 M to 2.3 ± 0.2. The enteric phases were simulated using NaCl 38.4 mmol/L, KCl 6.8 mmol/L, KH_2_PO_4_ 0.64 mmol/L, MgCl_2_(H_2_O)_6_ 0.33 mmol/L, NaHCO_3_ 85 mmol/L, CaCl_2_ 0.37 g/L, pancreatin 0.9 g/L, and bile 6 g/L (Sigma Aldrich). The pH was adjusted with NaOH 1 M or HCl 1 M to 5.0 ± 0.3 and 7.0 ± 0.3 for the enteric phases I and II, respectively.

The first bioreactor was fed 190 mL of the synbiotic açai juice for 10 min. The bioreactors were loaded with 100 mL, 60 mL, and 50 mL of gastric, enteric I, and enteric II simulated solutions, respectively. Each digestion phase lasted 2 h. The residence time in the bioreactor was 60 min with a 60 min transfer, achieved through automatic peristaltic pumps equipped with a mass flow controller. A volume of 280 mL was transferred to the second reactor (enteric I) and 298 mL to the third reactor (enteric II). Samples in each digestion phase were collected to evaluate the viability of *B. breve*, gluco-oligosaccharides, and dextran integrity [22].

### 2.4. Fecal Samples

The experiment adhered to the guidelines set forth by the Brazilian National Health Council (Resolution No. 466, 2012) and was approved by the Research Ethics Committee (CAAE: 56171022.2.0000.5054) of the Federal University of Ceara. Four healthy volunteers between the ages of 25 and 40 who had not consumed prebiotics, probiotics, or antibiotics for at least three months before donation, provided fresh human feces. Donors 1 and 2 provided fecal samples for fermentation with the juice sample at the beginning of the cold storage (day 0), while donors 3 and 4 provided samples for in vitro colonic fermentation at the end of the storage period (day 42). The fecal samples were collected in sterile universal collectors and homogenized in a buffered saline solution containing 0.1 M phosphate (5.8 g/L of NaCl +14.15 g/L of Na_2_HPO_4_ + 11.95 g/L of NaH_2_PO_4_) at pH 7.0. An inoculum suspension of 10% (*w*/*v*) of human feces was bubbled with sterile N_2_ to eliminate O_2._ Fecal fermentation was conducted at the beginning and end of the storage period. Given that beneficial changes in the microbiota were observed at the beginning (day 0) and end (day 42) of the storage period, these changes were expected to occur throughout the entire storage period since the microbial viability was assessed every 7 days to ensure that the synbiotic juice contained sufficient probiotics and prebiotics to maintain its potential benefits until the end of its shelf life. 

### 2.5. Fecal Fermentation

The fecal fermentation of synbiotic açai juice was conducted on the first day (0 h) and after 42 days of cold storage. The fecal inoculum 1% (*w*/*v*) was added to the bioreactor during the last digestion phase. Samples were collected after 0 h, 24 h, and 48 h of fermentation and centrifuged under 10,000× *g* at 4 °C/20 min. The supernatant was used to measure sugars, dextran, organic acids, and SCFA, and the solid phase (microbiota) was assessed using 16S rRNA sequencing [10,22].

### 2.6. Dextran Quantification

The dextran was precipitated by adding 3 mL of ethanol 96% (*v*/*v*) to 1 mL of the sample. The supernatant was used to quantify gluco-oligosaccharides, simple sugars, organic acids, and SCFA. Dextran pellet (precipitate) was resuspended in distilled water and quantified using the phenol–sulfuric method [26].

### 2.7. Analysis of Gluco-Oligosaccharides Degree of Polymerization Using Thin Layer Chromatography and Densitometry

The samples were diluted in ultrapure water (Milli-Q System, Millipore^®^, Burlington, VT, USA) and separated using thin layer chromatography (TLC) silica gel plates (Merck^®^, Darmstadt, Germany) 20 × 20 cm, Art. 1.05553.0001. An ATS4 (CAMAG^®^, Muttenz, Switzerland) was used to apply the samples (5 µL) to the TLC plates preconditioned at 60 °C, 1.0 cm from the plate border and at a 1.0 cm inter-sample distance. The plates were developed in the developing chamber using a mix of acetonitrile, ethyl acetate, 1-propanol, and water (8.5: 2.0: 5.0: 9.0, *v*/*v*/*v*/*v*) to separate the carbohydrates into two plate ascensions.

A solution composed of 0.3% (*w*/*v*) of 1-naftiletilenodiamine and 5% (*v*/*v*) of concentrated H_2_SO_4_ in methanol revealed the carbohydrates on the plate. The gluco-oligosaccharides were quantified using a TLC scanner 4 densitometer (CAMAG). Readings were performed at 490 nm. The Planar WinCATS Chromatography Manager software (Version 1.4.9.2001) was used to handle the data. Results were expressed as relative concentration (%), calculated as follows:

$$Relative\;concentration\left(\%\right)=\frac{A_{DPi}}{A_{T}}\times 100$$

where *A_DPi_* is the chromatographic area of an individual DP (*i* = 3 to 10) and *A_T_* is the sum of the individual chromatographic areas of all DP ${(\Sigma }_{3}^{10}A_{DPi}$).

### 2.8. Sugar, Organic Acids, and SCFA Determination

The simple sugars, organic acids, and SCFA were evaluated using high-performance liquid chromatography (HPLC) in an Infinity 1260 Agilent^®^ system (Agilent, Santa Clara, CA, USA). A refraction index detector at 35 °C was used to detect the sugars. The separation was achieved using a Supelco Ca column (300 × 7.8 mm) at 80 °C. Ultrapure water was the mobile phase at 0.5 mL/min. A UV-DAD 210 nm was used to detect the organic acids (ascorbic, acetic, and lactic acids) and SCFA (propionic, butyric, and isobutyric acids). The compounds were quantified using a calibration curve. The separation was achieved using a BIORAD HPX-87H column (300 × 7.8 mm) (BIORAD, Hercules, CA, USA) at 65 °C. An H_2_SO_4_ 5 mM solution was used as the mobile phase at 0.6 mL/min.

### 2.9. DNA Extraction through 16S rRNA Gene Sequencing

According to the manufacturer’s instructions, the bacterial DNA was extracted from the fecal fermentation samples (250 mg) using the DNeasy^®^ PowerSoil^®^ Pro Kit (QIAGEN, Hilden, Germany). The DNA integrity and concentration were evaluated through protein concentration using a NanoDrop^®^ spectrometer (Thermo Fischer Scientific, Waltham, MA, USA). After extraction, the samples were stored at −20 °C. The 16S rRNA gene sequencing was achieved using MiSeq Illumina (Illumina Inc., San Diego, CA, USA). A specific primer (515F-Y and 806R) (Illumina Inc., San Diego, USA) was used to sequence the V4 regions of the bacterial 16S rRNA gene according to the protocol described by Pereira et al. [27].

The DADA2 bank was used to generate taxonomic affiliations. The α-diversity was analyzed using the EstimateS software (Version 9.1.0) to obtain the species richness through the Chao1 index [28] and the Shannon diversity index [29].

### 2.10. Statistical Analysis

The results are expressed as mean ± standard deviation. A principal component analysis (PCA) was performed to show the correlation matrix among substrate consumption, metabolites, and fecal microbial species through the Origin software (version OriginPro 2024) (OriginLab Corporation ^®^, Northampton, MA, USA). A value of *p* < 0.05 was considered to be statistically significant.

## 3. Results and Discussion

### 3.1. Synbiotic Açai Juice: Production by Fermentation and Storage 

**Figure 2:**
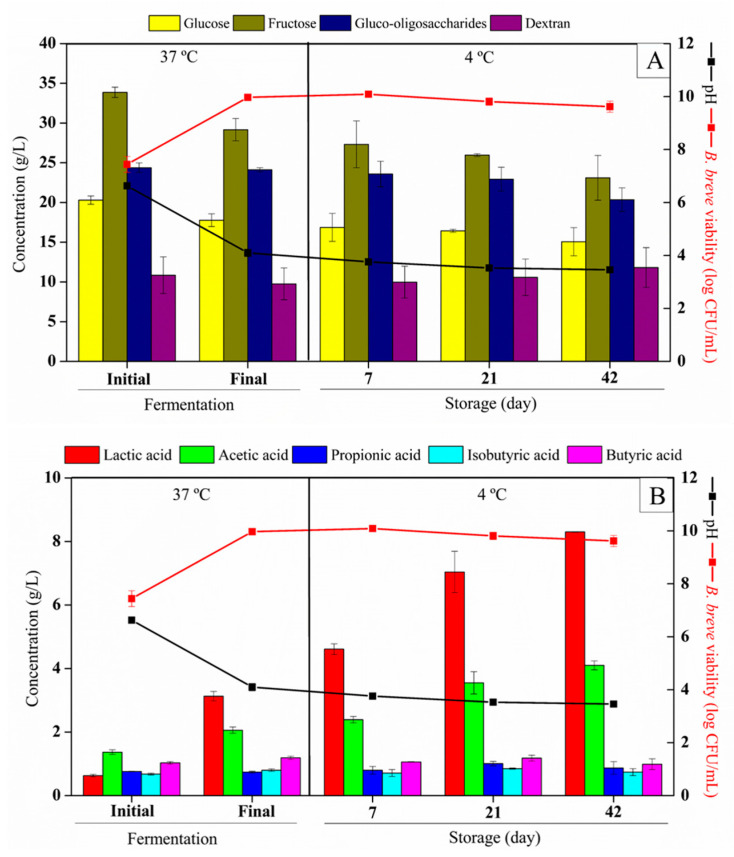
Microbial viability, pH, and carbohydrate concentrations during the fermentation and cold storage (**A**); lactic acid (g/L), SCFA (g/L), sugars (g/L), pH, and *B. breve* NRRL B-41408 viability in the potentially synbiotic açai juice before (0 h) and after fermentation (22 h) at 37 °C and during storage at 4 °C (days 7 to 42) (**B**).

The dextran and gluco-oligosaccharide concentration in prebiotic açaí juice (non-fermented) was 10.85 g/L and 8.13 g/100 mL, respectively. The gluco-oligossachride degree of polymerization (DP) ranged from 3 to 10. According to WHO/FAO and EFSA, 25 g is the maximum recommended daily fiber intake for an adult consumer [30,31]. The prebiotic juice fermented by *B. breve* resulted in a synbiotic açai juice with a cell viability of 9.97 log CFU/mL (2 log cycles increase) after 22 h of fermentation at 37 °C. The initial pH of the prebiotic açai juice was adjusted to 6.5, since the optimum pH for *Bifidobacterium* growth ranges from 6.0 to 7.0 (Figure 2) [32]. After fermentation, the pH decreased from 6.63 ± 0.07 to 4.10 ± 0.15 (*p* > 0.05). Figure 2 shows the microbial viability, pH, carbohydrates, and organic acid concentration in the potentially synbiotic açai juice before and after the fermentation with *B. breve* (22 h/37 °C) and during the cold storage (4 °C).

**Figure 3:**
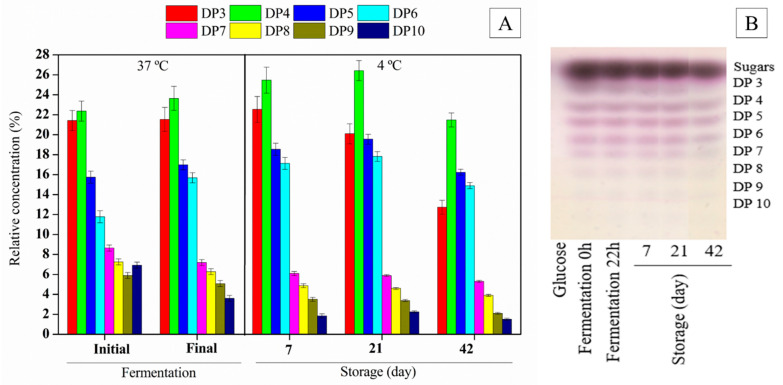
Relative concentration of each degree of polymerization (DP) of gluco-oligosaccharides (**A**) and degree of polymerization (DP) of oligosaccharides in a thin layer chromatography plate (**B**) from potentially synbiotic açai juice before (0 h) and after fermentation (22 h) at 37 °C and during the storage at 4 °C (days 7 to 42).

Bifidobacteria are known for their unique metabolic pathway called the bifid shunt or fructose 6-phosphoketolase pathway. This pathway helps them metabolize hexoses and produce key enzymes like fructose-6-phosphoketolase [33]. *B. breve* showed a preference for consuming glucose (12.46%) and fructose (13.87%) over other carbon sources like dextran and oligosaccharides (Figure 2A). A slight decrease in the relative concentration of the oligosaccharides with higher degrees of polymerization (DP7, DP8, and DP10) and an increase in DP6 indicate the hydrolysis of longer DPs into shorter-chain gluco-oligosaccharides, as observed by Leite et al. [14] (Figure 3).

The initial SCFA concentrations in the juice were 1.37 g/L, 0.76 g/L, 0.68 g/L, and 1.03 g/L of acetate, propionate, isobutyrate, and butyrate, respectively. At the end of the fermentation process (22 h), lactate and acetate concentrations increased by 400% and 50.36%, respectively, reaching 3.13 g/L and 2.06 g/L, respectively (Figure 2A). This result is consistent with the pH reduction. *B. breve* fermentation slightly increased the isobutyrate and butyrate concentrations (Figure 2B). SCFA and lactate are primary metabolites that can affect the composition and function of human microbiota [34]. Although lactate does not belong to the group of SCFA, it is produced by *Bifidobacterium* sp., which are lactic acid bacteria. However, under normal conditions, lactate might not accumulate in the colon due to the presence of some bacterial species, e.g., *Eubacterium hallii*, that can convert lactate into different SCFA [35]. 

Probiotic health benefits are dose-dependent, and the recommended daily dose is 10^9^ CFU of viable cells per dose [36]. Processing conditions, food matrix, pH, storage temperature, and dissolved oxygen are some factors that affect probiotic bacteria’s viability [33,37]. Tracking their viability rates within a specific shelf life is recommended to ensure adequate probiotic counts [38,39].

Maintaining the viability of *B. breve* in synbiotic açai juice during storage poses a significant challenge due to the low pH levels. Nevertheless, despite the presence of organic acid in its dissociated form, viable cell counts of *B. breve* displayed an increase of 64.5% up to the 15th day of storage at 4 °C. However, after day 21, cell viability decreased but remained above 8 log CFU/mL, an adequate value for fermented products containing probiotics. This means that 100 mL of synbiotic açai juice would provide a dose of 10^10^ CFU, which is higher than the minimum recommended dose. The results indicate that the cold storage of synbiotic açai juice can maintain an adequate viable cell count of *B. breve* for at least 42 days. This finding is relevant for those seeking to promote a healthy gut and underscores the potential of synbiotic açai juice as a dietary supplement rich in probiotics. At the end of the cold storage period (42 days), *B. breve* consumed fructose (20.78%) and gluco-oligosaccharides (15.59%). Lactate (8.3 g/L) and acetate (4.10 g/L) were produced during the storage period, and a reduction in pH was observed in the potentially synbiotic açai juice, indicating that *B. breve* maintained its metabolism even at low temperatures. The pH of the juice slightly decreased after the first week of refrigerated storage and remained constant until the end of the storage period, with a final pH value of 3.49 (Figure 1). The concentration of the other SCFA did not change during the cold storage.

Although acidity is a harmful condition for the survival of *B. breve*, the prebiotic oligosaccharide and dextran may have a protective effect on probiotic cells [7]. Furthermore, storing the probiotic cells at a low temperature (4 °C) may also contribute to their viability maintenance owing to the slow microbial metabolism. Kaewarsar et al. [40] suggest that synbiotic products should be kept at 5 ± 3 °C to prolong their shelf life. These findings are consistent with the study conducted by Saarela et al. [41] which reports that the viability of *B. animalis* subsp. lactis E2010 in fruit juices (orange, grape, and passion fruit) was significantly lower when stored at 20 °C compared to 4 °C for 6 weeks.

However, the viability of bifidobacteria during storage depends on the food matrix and the microbial strain. Guo et al. (2024) [42] found a survival rate of approximately 50% over a 30-day storage period (4–8 °C) with fermented milk beverages containing *B. adolescentis* B8589. Also, adding *B. adolescentis* improved the survival of *L. paracasei* PC-01 during milk co-fermentation. Similarly, the viability of *Bifidobacterium animalis* subsp. lactis BB-12 in orange juice decreased after 4 weeks of refrigerated storage (4 °C) [37].

Research has shown that including prebiotics in fermented products can enhance the survival of bifidobacteria. Prebiotics can further bolster microbial viability by providing a protective effect when combined with probiotics. Additionally, prebiotics serve as a source of nourishment for probiotic bacteria, fostering their growth. In support of this claim, a study by Kaewarsar et al. [40] yielded similar results.

Another study by Hesam et al. [43] reveals that pomegranate juice, fermented with *B. animalis* and enriched with prebiotics, displayed diminished viability after 10 days of storage. However, the synbiotic juice maintained an acceptable level of viability (10^6^ CFU/mL) even after 30 days of storage.

### 3.2. In-Vitro-Simulated Digestion

**Figure 4:**
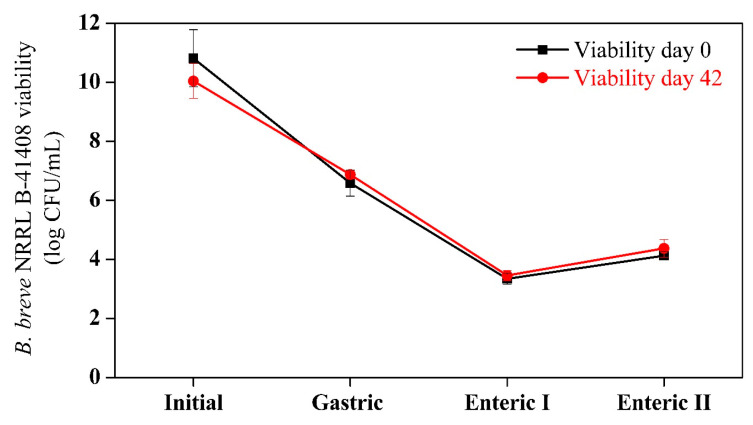
pH and *B. breve* NRRL B-41408 viability in synbiotic açai juice during the simulated digestion. Day 0 is the sample just after the fermentation, and day 42 is the sample at the end of the cold storage period.

The survival and settlement of probiotic bacteria in the gut tract’s epithelium are essential for their positive impact on health. In this study, the resistance of *B. breve* to gastrointestinal conditions was evaluated after fermentation (day 0) and at the end of the cold storage period (day 42), as shown in Figure 4. The results show no significant difference in microbial viability during the simulated digestion either at the beginning (day 0) or at the end (day 42) of the cold storage period.

The viability of *B. breve* decreased during the simulated gastrointestinal digestion phases for both D0 and D42 samples. However, it was able to resist the harsh conditions of the gastrointestinal tract and reach the final portion of the simulated duodenum phase with 4.12 log CFU/mL and 4.37 log CFU/mL for D1 and D42, respectively (as shown in Figure 3). This is significant because it indicates that the product could be successfully commercialized with extended cellular viability during refrigerated storage and still maintain its functionality when ingested. Similar results were observed by Cielecka-Piontek et al. [44], who also saw a reduction in the viability of *B. breve* DSM 16,604 and *B. animalis* subsp. lactis during the in-vitro-simulated digestion of chocolate. However, the authors did not report any viability recovery even when the conditions were more favorable, such as an increased pH in the following digestion phases. A synbiotic Sapota-of-Solimões juice was developed by Silva et al. [7], who found that *L. casei* was able to survive in the gastrointestinal tract during the enteric I (pH 5) and II (pH 7) phases. In contrast to the performance of *B. breve* in the synbiotic açai juice, *L. casei* in the synbiotic Sapota-of-Solimões juice exhibited a degree of viability recovery when subjected to an elevated pH in the enteric phase II. These findings suggest that the strain and food matrix significantly impact cell viability. 

This study confirms that cold juice storage does not affect the functionality of *B. breve*, as there was no significant difference in survival after in vitro digestion between day 0 and day 42. These data are crucial because the product can extend cellular viability significantly during storage and simulated digestion. After the simulated digestion, Langa et al. [45] evaluated *B. breve* viability in sheep’s milk cheese and obtained similar results. After 28 days of cold storage, the probiotic viability after the simulated digestion was 4.97 ± 0.40 log CFU/g, and, after 60 days of cold storage, the cell viability was only 1.74 ± 2.02 log CFU/g. 

**Figure 5:**
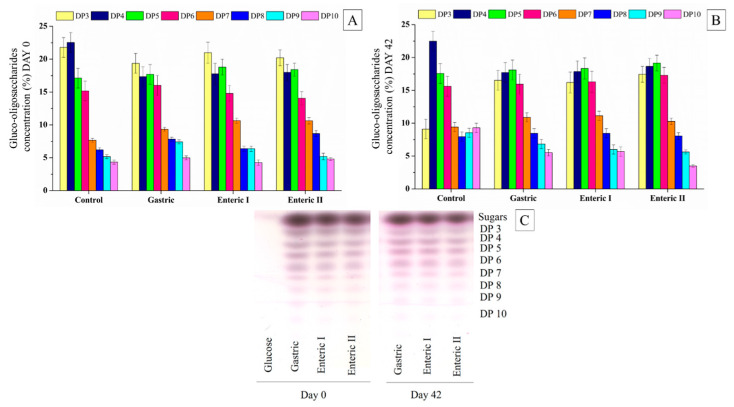
Relative concentration (%) of polymerization degree of gluco-oligosaccharides in the synbiotic açai juice during the in-vitro-simulated digestion on days 0 (**A**) and 42 (**B**) of the cold storage and degree of polymerization (DP) of gluco-oligosaccharides in the thin layer chromatography plate (**C**).

Gluco-oligosaccharides, dextran, and reducing sugars (glucose and fructose) were monitored during the in-vitro-simulated digestion. Figure 5 shows the degree of polymerization (DP) of oligosaccharides in the synbiotic açai juice during each digestion phase. The control was the non-digested synbiotic juice. The gluco-oligosaccharides were resistant to the gastrointestinal conditions, confirming the integrity of those complex carbohydrates after digestion from the beginning to the end of the storage period. Dextran was also resistant to gastrointestinal conditions during the cold storage, reaching 13.5 ± 1.07 g/L at the end of the in-vitro-simulated digestion. After digestion, orange juice containing gluco-oligosaccharides and dextran has been found to exhibit a similar behavior [22]. The resistance of dextran to gastric acid may be due to α-(1→6) glycosidic linkages [46]. Thus, dextran may also reach the colon and support the growth of beneficial bacteria.

### 3.3. Human Fecal Microbiota Fermentation

#### 3.3.1. Sugar Consumption 

**Figure 6:**
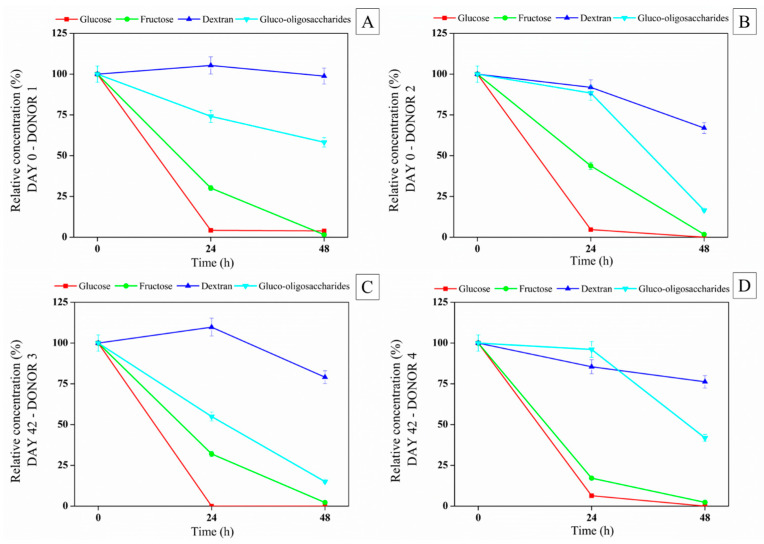
Relative concentration (%) of glucose, fructose, dextran, and gluco-oligosaccharides during in vitro colonic fermentation of the digested synbiotic açaí juice ((**A**–**D**) donors 1, 2, 3 and 4) on days 0 and 42 of the cold storage.

As shown in Figure 6, the fecal human microbiota consumed preferentially simple sugars (glucose and fructose), followed by gluco-oligosaccharides and dextran. The carbohydrate consumption pattern was the same for the juice at the beginning and end of the storage period and for all fecal samples. The gut microbiota of donors 2, 3, and 4, after 48 h of fermentation, consumed more than 50% of the gluco-oligosaccharides present in the synbiotic açai juice on days 0 and 42 of storage.

Dextran was the least consumed substrate, probably due to its higher molecular weight. After 48 h of in vitro colonic fermentation, dextran consumption was as follows: on day 0—donor 1 = 1.2% and donor 2 = 33.04%; on day 42—donor 3 = 20.92%; donor 4 = 23.74%. The colon’s resident microorganisms can distinguish between different types of carbohydrates. For example, dextran from *L. mesenteroides* NRRL B-1426 promotes the growth of *B. infantis*, *B. animalis* spp. lactis, and *L. acidophilus*, as reported by Kothari et al. (2015). In addition, Kim et al. [47] found that when probiotics ferment dextran, they produce large amounts of propionic acid and other metabolites. After 48 h of fermentation, the gluco-oligosaccharides had been significantly consumed (donor 1 = 41.77%; donor 2 = 83.52%; donor 3 = 84.99%; donor 4 = 58.15%). These compounds are essential for the growth of specific probiotic bacteria, particularly bifidobacteria [48].

#### 3.3.2. Organic Acids and SCFA Production

**Figure 7:**
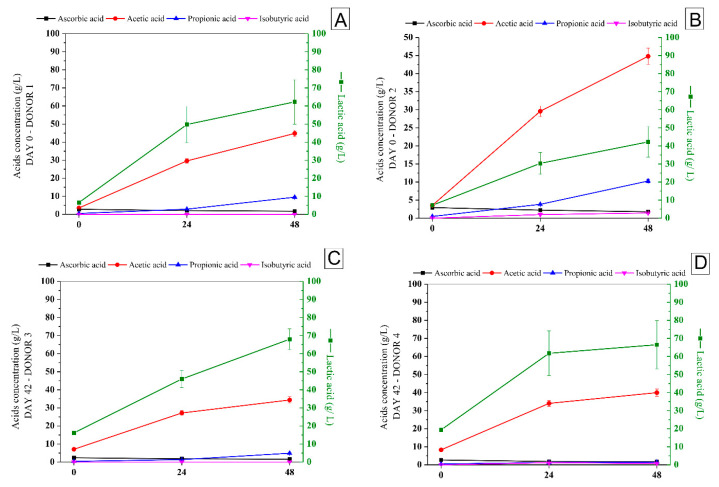
The concentration of organic acids and SCFA (g/L) during the fecal fermentation of the digested synbiotic açaí juice at the beginning (**A**,**B**) and end (**C**,**D**) of the cold storage period.

Figure 7 shows the production of organic acids and SCFA after 24 h and 48 h of fecal fermentation.

The main acids produced during the fecal fermentation of the synbiotic açai juice were lactic and acetic acids, followed by propionic and isobutyric acids (Figure 7). As expected, different concentrations were produced by the microbiota from the different donors due to the different initial composition (Figure 7), lactic and acetic acids being the major ones. Just after 24 h of fecal fermentation, the lactic acid production in the fecal fermentation samples from donors 1 and 2 experienced a 10- and 14-fold increase, respectively (juice storage day 0). The acetic acid experienced a 10- and 12-fold increase in fecal fermentation from the microbiota of donors 1 and 2. In contrast, propionic acid concentrations increased after 48 h of fecal fermentation at the beginning (donors 1 and 2) and end of the juice storage period (donor 3).

SCFA play an important role in gut barrier regulation, preventing the transit of pathogens or bacterial endotoxins, modulation of the immune system’s inflammatory response, the activation of the gut-brain axis, and the development of cardiovascular diseases [49]. Pan et al. [50] associated the production of propionic and acetic acids with the higher bifidobacteria population after 48 h of fecal fermentation of exopolysaccharide from *Leuconostoc pseudomesenteroides*. 

The fermentation products of some species are substrates for fermentation or are incorporated as intermediate metabolites into the metabolic pathways of other species, resulting in subsequently fermented substrates. This relation is known as cross-feeding, where the metabolic potential of the interacting members of a community can result in cross-feeding for mutual benefit [51]. Lactate, ethanol, and pyruvate decrease by subsequent bacterial utilization and SCFA production. The final products of sugar catabolism are SCFA, acetate, propionate, and butyrate, which account for 85–95% of total SCFA in all colon regions. Other fermentation end products, such as caproate and valerate, occur in lower amounts [35]. Also, SCFA, mainly acetic and propionic acids, inhibit inflammatory factors [52].

#### 3.3.3. Relative Abundance 

**Figure 8:**
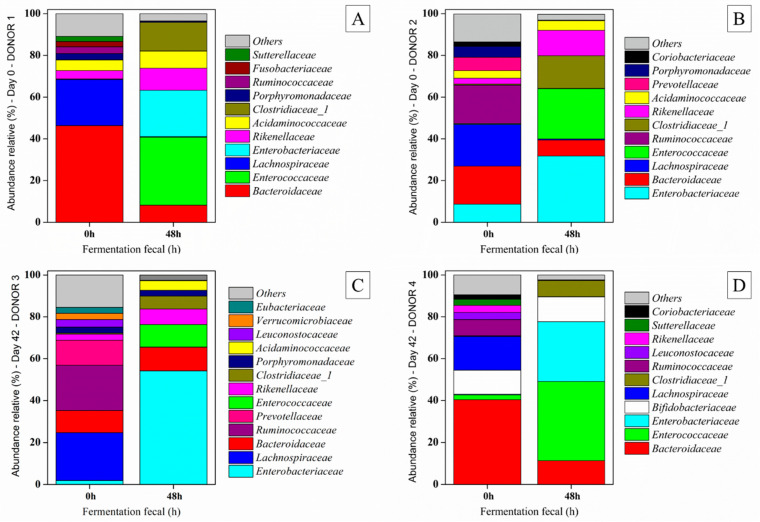
Relative abundance at the taxonomic family level before (0 h) and after (48 h) in vitro colonic fermentation. The data are relative to the fecal samples from two donors on day 0—the beginning of the cold storage period (donors 1 (**A**) and 2 (**B**)—and two donors (donors 3 (**C**) and 4 (**D**)) at the end of the cold storage period.

The synbiotic açai juice’s effect on human fecal microbiota was studied using 16S rRNA sequencing after 48 h of in vitro fermentation. The fecal microbiota was mainly composed of Firmicutes, Bacteroidetes, and Proteobacteria at the phylum level. These phyla play critical roles in gut health through metabolic functions such as SCFA production and immune regulation. Figure 8 shows the family relative abundance before and after the fecal fermentation of the synbiotic açai juice at the beginning of the cold storage period (donors 1 and 2) and at the end (donors 3 and 4). 

As expected, the microbiota from different donors exhibited a different composition, as shown in Figure 8. Before the fecal fermentation, donor 1’s microbiota predominately included the Bacteriodace and Lachnospirace families. After the fecal fermentation with the synbiotic açai juice, the relative amount of Bacteriodace and Lachnospirace decreased, while Enteroccaceae and Enterobacteriaceae were the predominant families (Figure 8A). 

Enterobacteriaceae is a family of bacteria commonly associated with beneficial and pathogenic roles in the human gut. Under normal conditions, certain species within this family can contribute to gut homeostasis by supporting the digestion of complex carbohydrates and SCFA production. However, an overgrowth of Enterobacteriaceae has been associated with dysbiosis and various gastrointestinal disorders, such as inflammatory bowel disease [53,54].

Despite the different composition of the donor 2’s microbiota (Figure 8B), after the fecal fermentation with the same beverage (synbiotic açai juice at the beginning of the cold storage period), the major families found in the microbiota of the donor 2’s sample were also Enteroccaceae and Enterobacteriaceae. Comparing Figure 8A,B, a very close relative amount of Enteroccaceae, Enterobacteriaceae, Bacteriodace, Closdridiace_1, and Rikenellacaceae was found in the microbiota from both donors. These findings suggest that synbiotic açai juice consumption may have a modulatory effect. After fecal fermentation, the relative abundance of the Lachnospiraceae family decreased, with values ranging from 0.13% to 0.5%. This trend was also observed by Leite et al. [22] after fecal fermentation of orange juice containing gluco-oligosaccharides and dextran.

The Clostridiaceae family belongs to the phylum Firmicutes. In the human gut, Clostridiaceae members can ferment dietary fibers and other complex carbohydrates that are not digested by the host, producing SCFA such as acetate, propionate, and butyrate, as shown in Figure 8. Clostridiaceae bacteria also regulate intestinal homeostasis [55]. *Clostridium cluster* XIVa (Lachnospiraceae family) can contribute to the microbiota’s colonization resistance against drug-resistant pathogens by converting primary bile acids into secondary bile acids [56,57]. Reducing this family’s relative abundance might also have a positive effect. The Enterobacteriaceae family is a diverse group of bacteria commonly found in the human gut. Members of this family contribute to various physiological functions, including digestion, vitamin synthesis, and immune modulation. The Clostrideaceace family also ferments dietary fibers, producing SCFA that nourish colon cells and support the gut barrier function. Some species synthesize B vitamins and vitamin K, essential for human nutrition. Enterococci produce small peptides with antimicrobial properties belonging to the bacteriocin-producing group [58]. 

Before fecal fermentation (0 h), donor 3 presented a higher microbial diversity than donor 4. At the end of the fecal fermentation (48 h), the same pattern observed for the juice samples at the beginning of the cold storage was followed—with an increase in Enterobacteriaceae and Enterococcaceae and a decrease in Lachnospiracea and Bacteriodaceae.

The results suggest that synbiotic açai juice consumption influences gut microbial diversity, promoting families like Clostridiaceae. These families ferment dietary fibers to produce SCFA and synthesize essential vitamins, including B vitamins and vitamin K. These functions are critical for human nutrition, gut barrier integrity, and immune modulation. Although reducing Lachnospiraceae might weaken colonization resistance, the overall increase in SCFA production and other beneficial effects indicate that this beverage can potentially improve gut health. 

Further research is necessary to explore the long-term implications of such shifts in the gut microbiota, including the potential influence on gut permeability and immune function. The overall effects of synbiotic açai juice on gut health will depend on the relative abundance of specific bacteria and the functional outcomes of microbial metabolism. Therefore, while the increase in Enterobacteriaceae should be noted, the positive outcomes, such as increased SCFA production and improved microbial diversity, offer promising evidence of the potential health benefits associated with the consumption of this synbiotic beverage [49,59].

**Figure 9:**
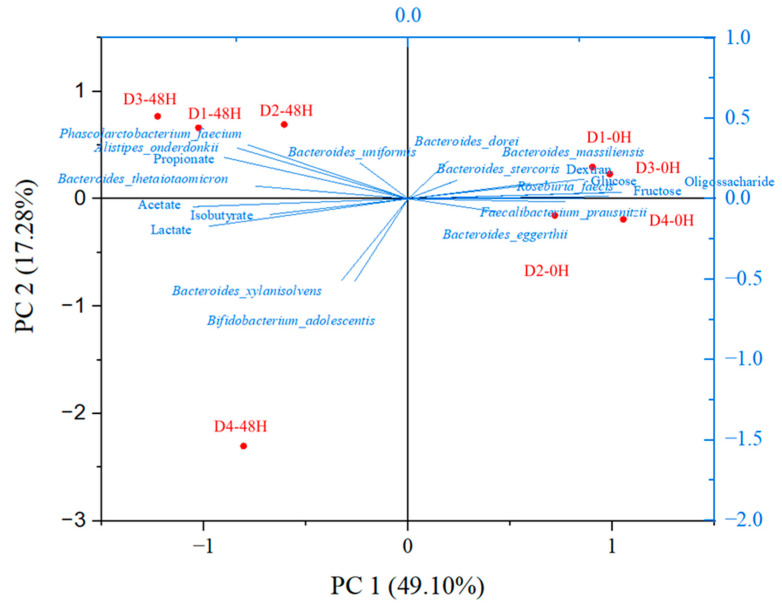
Principal component analysis of the effects of the digested synbiotic açai juice before (0 h) and after (48 h) in vitro colonic fermentation on fecal microbiota species from four donors (D1—donor 1, D2—donor 2, D3—donor 3, and D4—donor 4).

Principal component analysis (PCA) shows the influence of synbiotic açai juice on the microbial composition of human fecal samples. Figure 9 shows the scores, loading graph, and the main components, PC1 and PC2, used to classify the samples. PC1 and PC2 explained 49.1% and 17.28% of the total variance in the data (66.38%), respectively.

The PC1 shows the donors’ microbiota before fermentation (0 h) and the composition after fermentation (48 h), discriminating the microbiota according to fermentation time. High concentrations of lactate, acetate, propionate, and isobutyrate were correlated with the highest concentrations of *Phascolarctobacterium faecium*, *Alistipes onderdonkki*, *Bacteroides thetaiotaomicron*, *Bacteroides uniformis*, *Bacteroides xylanisolvens*, and *Bifidobacterium adolescentis*, after 48 h of fermentation.

In the PC2, high lactate, acetate, and isobutyrate concentrations correlated directly with *Bifidobacterium adolescentis*. These acids are the main metabolites of the intestinal microbiota, produced mainly by bifidobacteria [52]. Furthermore, high propionate concentrations directly correlated with the high abundance of Bacteroides species, such as *Bacteroides thetaiotaomicron*, *Bacteroides uniformis*, *Bacteroides xylanisolvens*, *Bacteroides dorei*, *Bacteroides stercoris*, and *Bacteroides massiliensis*. *Bacteroides* spp. are associated with propionate production [49]. SCFA are known for their anti-inflammatory effects and can, therefore, contribute to the prevention and treatment of diseases.

#### 3.3.4. Diversity and Richness

**Figure 10:**
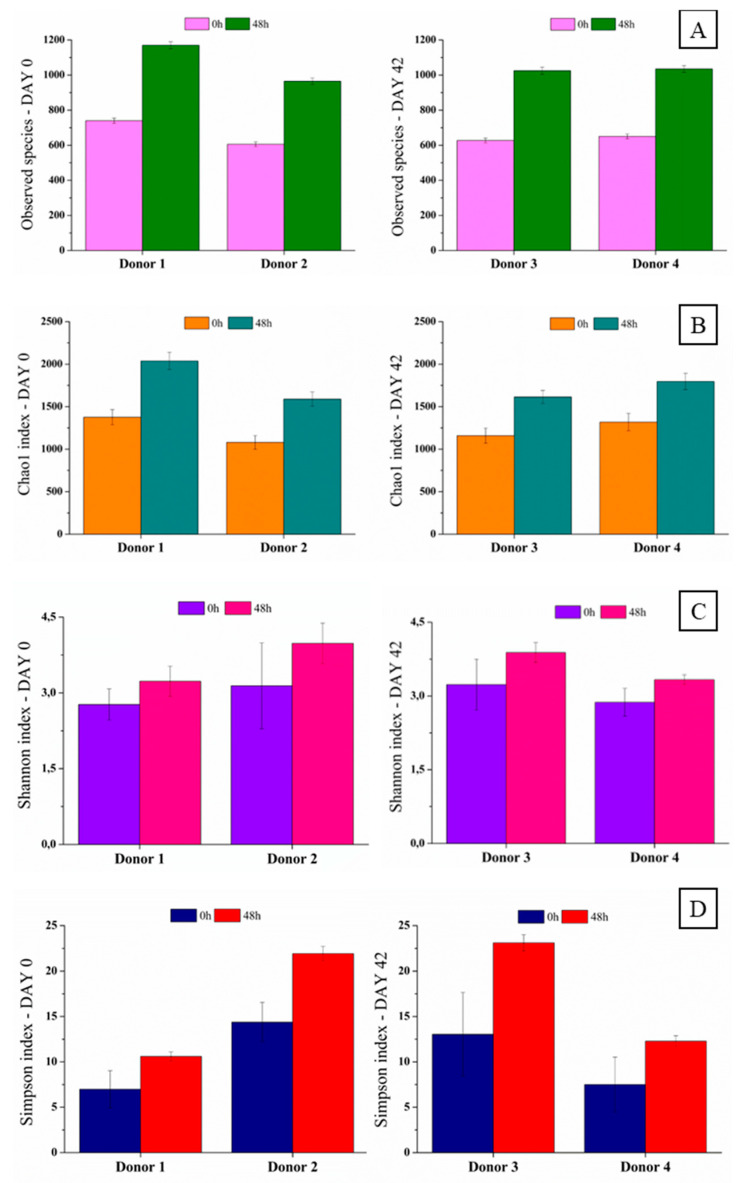
Observed species (**A**), Chao1 (**B**), Shannon (**C**), and Simpson’s (**D**) indexes of the fecal microbiota of the four donors (1,2,3, and 4) before and after fecal fermentation of the digested synbiotic açai juice on days 0 and 42 of the cold storage period. Donor 1 = day 0; donor 2 = day 0; donor 3 = day 42, and donor 4 = day 42.

Among the α-diversity indices, the observed species (Figure 10A) and Chao1 (Figure 10B) index mainly reflect the richness of the sample community. In contrast, the Shannon (Figure 10C) and Simpson’s (Figure 10D) indexes reflect the diversity of the sample community. The synbiotic açai juice increased the bacterial community, as indicated by the observed species value and Shannon index. According to the Shannon index, the fecal microbiota increased by 116.6% (donor 1), 125.15% (donor 2), 117.2% (donor 3), and 116.02% (donor 4) after 48 h of fermentation. The Simpson’s index also increased to 151.86% (donor 1), 152.5% (donor 2), 177.22% (donor 3), and 163.73% (donor 4) following fermentation. Shannon and Simpson indexes increased because of the relative abundance of the Enterobacteriaceae and Enterococaceae families (Figure 8) and the growth of Bacteroides species (Figure 9). Comparing the data among the four groups suggests that the richness and diversity of fecal microbial communities improved following consumption of the synbiotic açai juice.

After the fecal fermentation of the synbiotic açai juice during the cold storage period, the Shannon and Chao1 indexes increased for all donors. Human fecal microbiota diversity is associated with an inverse susceptibility to certain diseases [52,60]. For example, the species diversity index used on patients with inflammatory bowel disease was smaller than its counterpart among healthy humans [61,62,63]. Thus, ingesting synbiotic açai juice may improve the diversity of the microbiota and contribute to the gut microbiota balance.

## 4. Conclusions

This study demonstrates that açai juice is a suitable matrix for developing a synbiotic beverage, maintaining *Bifidobacterium breve* viability above 8.0 log CFU/mL after fermentation and 42 days of cold storage. The synbiotic juice containing gluco-oligosaccharides and dextran provided the necessary nutrients for the growth of *B. breve* NRRL B-41408. These findings indicate that the açai juice is a viable non-dairy option for carrying probiotics. During cold storage, the probiotics do not consume gluco-oligosaccharides and dextran. These potential prebiotic compounds are resistant to gastrointestinal conditions after fermentation and after 42 days of cold storage, maintaining the prebiotic characteristics throughout the juice’s shelf life. The juice induces changes in gut microbiota composition, including an increase in Enterobacteriaceae and Enterococcaceae species and a decrease in Lachnospiraceae species. While these shifts require further investigation, the overall increase in microbial diversity and SCFA production highlights its potential to improve gut health. Future research should focus on validating these effects in vivo, optimizing the synbiotic formulation, and exploring the functional roles of specific microbial shifts. Synbiotic açai juice holds promise as a functional food that promotes gut health and microbiota balance.

## Data Availability

The original contributions presented in this study are included in the article. Further inquiries can be directed to the corresponding author.
